# Analysis of Porosity–Water/Binder Index for Prediction of Strength and Stiffness of Cemented Sands: A Comparative Study

**DOI:** 10.3390/ma18020268

**Published:** 2025-01-09

**Authors:** Jesús Alberto Alcalá Vergara, Luis Carlos Suárez López, Yamid E. Nuñez de la Rosa, Oriana Palma Calabokis, Jair de Jesús Arrieta Baldovino

**Affiliations:** 1 Civil Engineering Program, Universidad de Cartagena, Cartagena 130015, Colombia; jalcalav@unicartagena.edu.co (J.A.A.V.); lsuarezl@unicartagena.edu.co (L.C.S.L.); 2 Faculty of Engineering and Basic Sciences, Fundación Universitaria Los Libertadores, Bogota 110231, Colombia; opalmac@libertadores.edu.co

**Keywords:** porosity–water/binder index, unconfined compressive, stiffness, artificially cemented, sands

## Abstract

Artificial sand cementation improves stability, stiffness, and mechanical strength, making it a critical process in geotechnical applications. This study analyzes the capability of the porosity–water/binding agent index $\left(\eta C_{w}/B_{iv}\right)$ to predict cemented sands’ unconfined compressive strength $\left(q_{u}\right)$ and stiffness $\left(G_{o}\right)$. Four Colombian sands, i.e., Luruaco, Medellín, Lorica, and Bogotá (stabilized with Portland cement), and were compared with three Brazilian sands: i.e., Osorio, Porto Alegre, and Rio Pardo were evaluated, stabilized with combinations of carbide lime and glass powder, using varying binder contents and a curing period of seven days subjected to ultrasonic pulse velocity (UPV) tests and unconfined compressive strength (UCS) tests. The results indicate that incorporating water content into the index significantly enhances predictive accuracy, achieving R^2^ values above 0.94 for Colombian sands and considerably better fits for Brazilian sands than the traditional porosity/binder index. This new alternative provides an appropriate parameter for representing the small-strain stiffness and unconfined compressive strength of artificially cemented sands stabilized with various types of binders. Furthermore, the new index proved to be more effective in predicting the behavior of uniform and loose-graded sands, such as those from Bogotá and Lorica, which rely more heavily on binder volume and water content to achieve greater strength and stiffness. Lastly, the predictive model, validated against experimental results, achieved reliability indices (R^2^) of 0.9791 for stiffness and 0.9799 for strength prediction.

## 1. Introduction

Artificial soil cementation for soils requiring improvements in strength and stiffness is an alternative that allows for the enhancement of local soils, thereby reducing costs in construction projects. Supplementary cementitious materials (SCM) are incorporated to reduce the embodied carbon emissions in the construction sector by partially or wholly replacing cement. Among the SCM studied are wood ash, fragments of polyethylene terephthalate (PET) bottles, crushed limestone waste (CLW), rice husk ash (RHA), and carbide lime (CL) [1,2,3,4]. Consoli et al. [5] investigated the potential of waste glass and carbide lime (CL) as a possible environmentally sustainable binder to improve the behavior of three different silica sands. They conducted unconfined compressive strength, ultrasound, and durability tests on cylindrical specimens with variations in ground glass (GG) content ranging from 20% to 30% and CL content from 3% to 11%. For each sand, dry-specific weights were determined for specimen preparation. The results indicated that GG particles interact with calcium in a hydrated alkaline environment, producing binding products such as calcium silicate hydrate gel, the same product formed from the hydration of Portland cement. Hence, both residues serve as a binder to replace Portland cement. In another study, Wang et al. [6] concluded that during NH_4_NO_3_ dissolution, the C-S-H gel undergoes a significant change in its average chain length (ACL), indicating an increase in polymerization due to calcium loss. This phenomenon is associated with the formation of longer bonds between silica tetrahedra, resulting in more polymeric structures within the C-S-H gel.

Additionally, Consoli et al. [7] investigated the potential of pozzolanic residues (coal bottom ash—BA, rice husk ash—RHA, ground glass—GG, and ground clay bricks—GCB) and carbide lime (CL) as binding agents in poorly graded sand. For the preparation of cylindrical specimens, the moisture content for BA and GG was 11%, while for RHA it was 14%. Different curing temperatures were employed for 7 days (23 ± 2 °C and 40 ± 2 °C). The pozzolana percentages used were 10%, 20%, and 30%, while the CL percentages were 8% for BA, 3%, 5%, and 7% for GG, and 5%, 8%, and 9% for RHA and GCB. The unconfined compressive strength, ultrasound, and durability tests demonstrated that pozzolanic materials combined with CL were effective as binding agents, representing significant sustainability advantages.

The rational dosage method, which relates to porosity (η) and volumetric cement content $\left(C_{iv}\right)$, key factors for determining soil strength, was established by Consoli et al. [8] as a way to achieve the desired strength by modifying the $\left(\eta /C_{iv}\right)$ index. Under this methodology, Consoli et al. [8] demonstrated the construction of a characteristic curve that can be used to achieve different soil strengths. This curve, which depends on the material type, defines how strength $\left(q_{u}\right)$ relates to variations in the porosity-to-cement ratio. The $\left(\eta /C_{iv}\right)$ index has been applied with binders other than Portland cement. Its use with lime has been extensively studied in various soil stabilizations; however, research on its application with pozzolans has shown slower progress, as pozzolans do not inherently act as cementitious materials and require activators for cementation. Based on this, the $\eta /B_{iv}$ (porosity/binder) has been developed, replacing the volumetric cement content with the total volumetric binder content, corresponding to the absolute volumes of lime, cement, and pozzolan. In general, results from various studies [9,10,11,12] indicate that an increase in the $\eta /B_{iv}$ index leads to a reduction in unconfined compressive strength. This effect is caused by increased porosity, which decreases particle interlocking, reducing frictional and chemical cementation strength due to the lower availability of binding agents for development.

For fine-grained cohesive soils, a high water-to-cement ratio enhances fluidity, making the mixing and compaction of the soil easier. Conversely, for coarse-grained non-cohesive soils, a low water-to-cement ratio should be adopted to prevent reductions in cohesion and water retention [13]. An improvement in the pore structure and compactness of the soil-cement microstructure occurs with an increase in the cement content and the use of ultrafine cement (UFC). This is due to the hydration spaces that create macropores and the action of cementitious products, which continuously bond soil particles and fill the pores [14]. A prolonged hydration time can reduce the impact of temperature on the hydration products [15].

Beyond the applied relationship, authors have demonstrated the need to use an exponent x that modifies the volumetric binder content $\left(B_{iv}\right)$, improving the fit of the proposed correlations. When the exponent x=1, porosity and cement content have an equivalent influence on soil-cement strength. For x<1, porosity becomes more relevant in the mix, while x>1 indicates that cementitious bonds significantly influence strength [16]. Furthermore, the index was not solely derived theoretically, as studies have demonstrated that its formulations are not exclusively based on empirical evidence. Research by Diambra et al. [17,18] on soils stabilized with cement and the study by Festugato et al. [19] on soils improved with fibers and cement have suggested the possibility of organizing empirical and theoretical equations based on the combined contributions of strength from the material mix. These equations were developed to describe the unconfined compressive strength of cement-treated soils, Consoli et al. [8]:

(1) $$q_{u}=A{\left[\frac{\eta }{{\left(C_{iv}\right)}^{x}}\right]}^{-C}$$

The parameter A in Equation (1) is of great theoretical importance, as it is influenced by the strength of cementation bonds and the sand matrix, with the latter being less influential than the cementation bonds [18]. The values of exponents C and x depend on the soil matrix. The parameter A is a multiplier resulting from the combination of soil matrix properties, cemented phase properties, and curing time. Similarly, Diambra et al. [17,18] and Festugato et al. [19] stated that A is predominantly calculated based on the matrix’s frictional strength and the cemented phase’s strength. Additionally, the exponent C depends on the soil matrix properties, which affect A.

The applicability of the $\left(\eta /{B_{iv}}^{x}\right)$ index has been corroborated in various studies [20,21,22], yet none have considered the additional factor of water content during soil stabilization. Baldovino et al. [23] studied the incorporation of water content into the $\left(\eta /{B_{iv}}^{x}\right)$ index to predict the strength, stiffness $\left(G_{o}\right)$, and durability under wetting-drying cycles of different artificially cemented soils. They concluded that applying the new parameter suits soil-lime, soil-cement, and geopolymerized soil mixtures. Additionally, they determined that water content does not affect the relationship between splitting tensile strength and compressive strength. Similarly, Baldovino et al. [24] applied the new parameter in a comparative analysis of the strengths of different stabilized soils, reaffirming its suitability for designing soil-lime, soil-cement, and geopolymerized soil mixtures.

This study aims to verify the applicability of the porosity–water/binder ratio in stabilizing seven sands—four from different open-pit mines in Colombia and three from Brazil—artificially cemented with two binding agents (Type III Portland cement for Colombian sands and lime with ground glass for Brazilian sands). Additionally, the study seeks to create equations for calculating unconfined compressive strength and stiffness. The binder content, specimens’ dry weight, and soil and binders’ specific gravity are unique parameters for developing these equations.

As a distinguishing factor from the studies by Baldovino et al. [23,24], this research applies the mentioned ratio to predominantly sandy soils. Additionally, it incorporates four Colombian sands that have not been previously studied under this index. The studies by Baldovino et al. [23,24] will serve as a comparative benchmark to verify the applicability of the new index in soils different from those analyzed by these authors. This is intended to be presented as an added factor to validate its descriptive applicability for the mechanical behavior of various soils.

## 2. Materials and Methods

### 2.1. Materials

**Figure 1:**
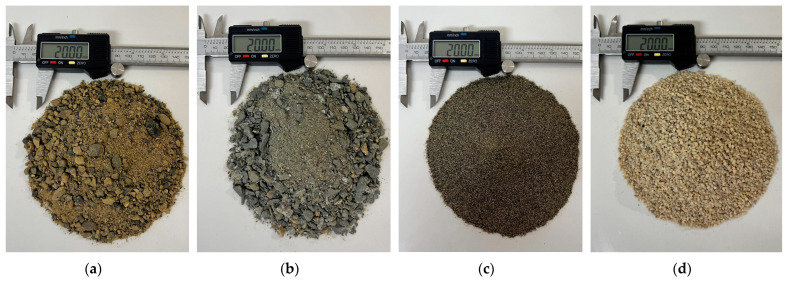
Sandy soil samples from Colombia: (**a**) soil sample from Luruaco; (**b**) soil sample from Medellín; (**c**) soil sample from Lorica; (**d**) soil sample from Bogotá.

He soils involved in the study were predominantly sandy and originated from Colombia (Figure 1) and Brazil. Lopez et al. [25] studied four sandy soils extracted from different open-pit mines in Colombia (Figure 1). The sands from Luruaco (Atlántico), Lorica (Córdoba), and Bogotá D.C. were classified as SP sands, while the sand from Medellín (Antioquia) was classified as SW. In this study, the authors used Type III Portland cement as a binding agent in contents of 3%, 5%, 7%, and 9%, and selected three different density points for each sand. Additionally, a water content of 10% and a curing time of 7 days were used.

On the other hand, the three sands from Brazil were studied by Consoli et al. [5], which included sands from Osorio, Rio Pardo (RP), and Porto Alegre (POA). In all cases, their classification under the Unified Soil Classification System (USCS) was poorly graded (SP). The moisture content applied to prepare the specimens was 11%, with a curing time of 7 days. The binders used resulted from pozzolanic reactions between silica in its amorphous phases (present in ground waste glass, GG) and Ca^++^ (present in carbide lime, CL) in an alkaline environment. GG contents of 20% and 30% were used for the three sands. For Osorio sand, CL contents of 3%, 5%, and 7% were applied, while for RP and POA sands, CL contents of 5%, 8%, and 11% were used.

Table 1 presents the results of the tests conducted to define the geotechnical characteristics of the studied sands, while Table 2 shows the percentages of the parameters involved in the tested specimen fabrication process. In both studies, only the porosity/binder index was applied without considering the water volume content.

### 2.2. Methodology

The methodology initially implemented in the research consists of the theoretical development of the porosity–cement index, followed by the semi-empirical implementation of the new porosity–water/binder index, where the added variable is the volume of water. Finally, an experimental study was conducted on artificially cemented soils, where the porosity–binder relationship has been previously used. However, it will now be studied using the index incorporating water content to demonstrate its efficiency in predicting strength and stiffness properties.

The properties of strength, deformability, and durability are directly influenced by the conditions under which soil improvements using cement are carried out. Lade and Trads [26] have studied the impact of stabilization processes on soil characteristics, identifying the three most influential factors in the stress–strain response and volumetric behavior of artificially cemented granular soils: effective confinement pressure, initial void ratio, and cement content. Additionally, they note that other significant factors include the physicochemical characteristics of the soil, soil texture, and moisture content during compaction. The latter factor is also emphasized by Horpibulsuk et al. [27], who mention that the volumetric water content plays an essential role in the stabilization process and should be considered. Moreover, Ríos et al. [28] state that the main factors governing the behavior of structured material are the cemented structure and the arrangement between grains, at least until a stress state is reached, in which the phenomenon of plasticization occurs.

#### 2.2.1. Specimen Molding and Preparation

**Figure 2:**
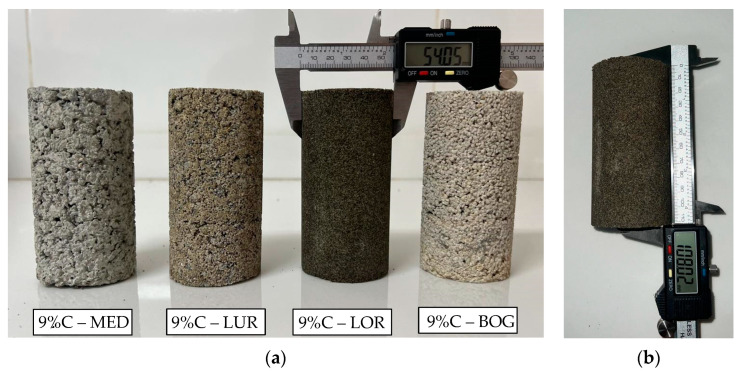
Samples of Colombian sands cemented with 9% Type III Portland cement on the sixth day of curing, demolded for mass and geometry measurements before saturation. (**a**) Specimens corresponding to Medellín, Luruaco, Lorica, and Bogotá, respectively; (**b**) measurement of specimen length.

Colombian sands were cemented using high early-strength Portland cement and prepared in molds with a diameter of 5.4 cm and a height of 10.8 cm, maintaining a 1:2 ratio. The remaining preparation parameters followed the methodology described by López et al. [25]. The masses of all 144 specimens were measured on the sixth day of curing, prior to saturation, to determine their densities and moisture content after being submerged for 24 h. A total of 36 molds were prepared for each type of sand, as shown in Figure 2.

#### 2.2.2. UCS and Stiffness (Non-Destructive) Program

**Figure 3:**
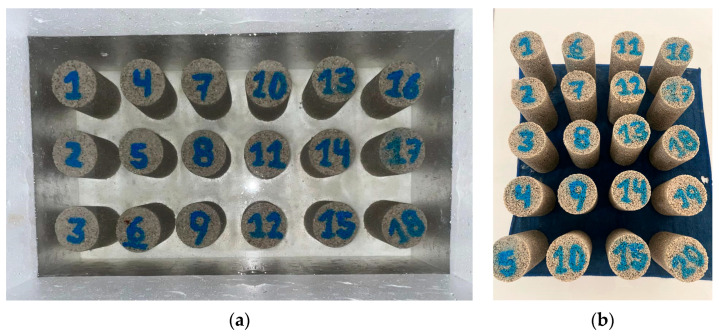
Final stage of the curing process for cemented specimens. (**a**) Saturation of soil specimens (Bogotá) and cement for approximately 24 h in a humid chamber. (**b**) Surface drying of the specimens following saturation.

The cemented specimens were subjected to a saturation process (Figure 3a) on the sixth day of curing to minimize the impact of matric suction on the samples, as indicated in previous studies [5,8,29,30,31,32]. Approximately 24 h later (for a total curing period of 7 days), the specimens were partially dried from the containers (Figure 3b), and their mass was re-recorded. Subsequently, an ultrasound test was performed to determine the small-strain shear modulus $\left(G_{o}\right)$ using the Pundit Lab equipment, Proceq (Schwerzenbach, Switzerland). This equipment determines the propagation velocity of compression waves (P-waves) and shear waves (S-waves) within the tested material [30]. The frequency used for P-waves was 54 kHz, while for S-waves it was 250 kHz.

**Figure 4:**
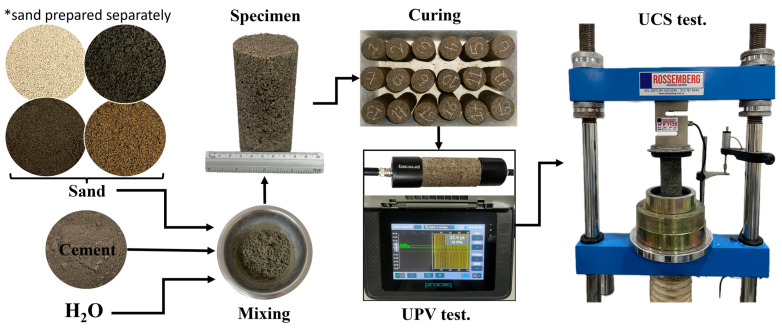
Graphical abstract of specimen preparation and tests conducted in this study: UPV (ultrasonic pulse velocity) and UCS (unconfined compressive strength). * Each specimen contains a percentage of a single type of soil; in other words, they are not mixed with each other.

After completing the stiffness test, the specimens were subjected to the unconfined compression test (UCS test), as shown in Figure 4. This test was performed in a 50 kN multi-test hydraulic press with a sensitivity of 0.1 kN, and the breaking load was applied at a rate of 1.00 mm per minute [25].

#### 2.2.3. Theoretical Derivation of the Porosity–Cement Index

Diambra et al. [17] assumed that the soil–cement mixture, artificially generated, behaves as an isotropic composite material, where failure is determined by superimposing the strength contributions of the cement and soil phases. Between these two phases, they assumed strain compatibility and simultaneous failure in both. In the cement phase, the strength was described using the Drucker–Prager failure criterion, while for the soil matrix, strength was represented by the relationship between mean stress and a state parameter in terms of material porosity (i.e., current porosity/critical state porosity). Equation (2) arises from the assumptions above.

(2) $$q_{u}=\frac{6\mu_{c}\sigma_{c}^{c}}{K_{c}\left(1-\beta \right)+3\left(\beta +1\right)}\left[\frac{K_{c}-M{\left(\frac{\eta_{cs}}{\eta }\right)}^{a}}{3-M{\left(\frac{\eta_{cs}}{\eta }\right)}^{a}}\right]$$

where $\mu_{c}=C_{iv}/100$. M is the critical state strength ratio of the soil; $\eta_{cs}$ is the critical state porosity (considered a constant for each soil); a is the parameter that governs the dependence of soil strength on its density; $\sigma_{c}^{c}$ is the compressive strength of the cement phase for a specific curing time; β is the ratio of uniaxial compressive strength to tensile strength of the cement; and $K_{c}$ is the cement stress ratio. Equation (2) is not similar to the empirical formula presented in Equation (1), as it is not a linear function of the soil’s maximum strength. However, Equation (3) can be introduced:

(3) $$\frac{K_{c}-M^{*}}{3-M^{*}}≅M^{*}\left(-0.6+0.45K_{c}\right)$$

Equation (3) is incorporated into Equation (2), and with further manipulation, a complete alignment between the theoretical and empirical parts can be achieved, as shown in Equation (4). Through this approach, the coefficients x and C in Equation (1) can be determined based on the properties of the soil matrix. Additionally, the scalar parameter B, which is the first term on the left-hand side of the equation, is influenced by the properties of the soil or the cement phase.

(4) 6Mσcc(−0.6+0.45Kc)ηcsa100[Kc(1−β)+3(β+1)][η(Civ)1a]−a=B[η(Civ)1a]−a

The effectiveness and accuracy of Equation (4) were demonstrated by Baldovino et al. [23], albeit without considering the volumetric water content.

#### 2.2.4. Semi-Empirical Derivation of the New Porosity–Water/Binder Index

The porosity–binder index $\left(\eta /B_{iv}\right)$ was introduced by Consoli et al. [8] and became a practical tool for evaluating the unconfined compressive strength of artificially cemented soils. The binder content $\left(B_{iv}\right)$ corresponds to a ratio obtained by dividing the cementing agent’s volume by the sample’s total volume, expressed as a dimensionless value. The unconfined compressive strength $\left(q_{u}\right)$ and the initial stiffness at small strains $\left(G_{o}\right)$ are functions of $\eta /B_{iv}$, adjusted with two empirical exponents (x y C) and an empirical constant $A_{q}$, expressed in kPa, as shown in Equation (5):

(5) $$q_{u}\;V\;G_{o}=A_{q}{\left[\frac{\eta }{{\left(B_{iv}\right)}^{x}}\right]}^{-C}$$

The scalar $A_{q}$ is governed by the properties of both the soil and the cementitious matrix. Therefore, Equation (5) is extended to investigate the strength, stiffness, and durability of different mixtures stabilized using cement and geopolymers. One limitation of this parameter is that it does not consider the volume of water within the mixture. Another limitation is the uncertainty regarding the range of its applicability, as strength tends to be infinite for minimal values of $\eta /B_{iv}$. Baldovino [23,24] proposes multiplying this index by $C_{w}/B_{iv}$, where $C_{w}$ is the total sample volume divided by the water volume. Thus, Equation (5) becomes:

(6) $$q_{u}\;V\;G_{o}=A_{q}{\left[\frac{\eta C_{w}}{{\left(B_{iv}\right)}^{2a}}\right]}^{-d}$$

Where a and d are empirical parameters that depend on the properties of the soil and the binder. This equation has already been applied by various authors to different soils using various binding agents, with the results obtained validating the incorporation of the $C_{w}$ parameter for the soils involved in those studies [23,24].

## 3. Results and Discussion

In this section, the methodology for rational dosing is applied and validated through the porosity–water/binder index (Equation (6)), based on the test results for unconfined compressive strength $\left(q_{u}\right)$ and stiffness measured by ultrasonic pulse velocity $\left(G_{o}\right)$ for the soils studied by [5,25]. These response variables were correlated with the porosity–water/binder index $\left(\eta C_{w}/B_{iv}\right)$ across all soil–binder mixtures presented in Table 2. The corresponding soils were geotechnically classified, as shown in Table 1. The study encompasses seven mixtures, four of which consist of artificially cemented sands from Colombia utilizing Portland cement as the binder. Additionally, three mixtures from Brazil were analyzed, incorporating glass powder and carbide lime as alternative binding agents.

The results will be analyzed in two main subsections to provide a structured and comparative understanding of the findings.

The first subsection focuses on applying the porosity–water/binder index to predict the unconfined compressive strength $\left(q_{u}\right)$ of compacted mixtures. This section explores the relationship between the index and the mechanical properties of each material, highlighting the predictive capability of the index based on experimental data.

The second subsection delves into stiffness $\left(G_{o}\right)$ predictions, evaluated through ultrasonic pulse velocity. This analysis includes a detailed comparison between the experimental values obtained using the new index proposed by Baldovino et al. [23,24] and the index previously developed [8,9,33,34,35,36,37,38]. This approach aims to identify similarities and differences in the accuracy of both indices, providing a critical perspective on their applicability under different conditions and for various materials.

The porosity–water/binder index $\left(\eta C_{w}/B_{iv}\right)$ has emerged as an effective tool for predicting the strength and stiffness of artificially stabilized soils, introducing water content as a key factor in characterizing the mechanical behavior of mixtures [1]. Unlike the traditional porosity–binder index $\left(\eta /B_{iv}\right)$ this new index allows for a more accurate correlation by directly accounting for the influence of water on the stabilization process, thus improving the prediction of unconfined compressive strength and initial stiffness [2].

### 3.1. Application of the Porosity–Water/Binder Index to Predict the Strength of Cemented Sands

**Figure 5:**
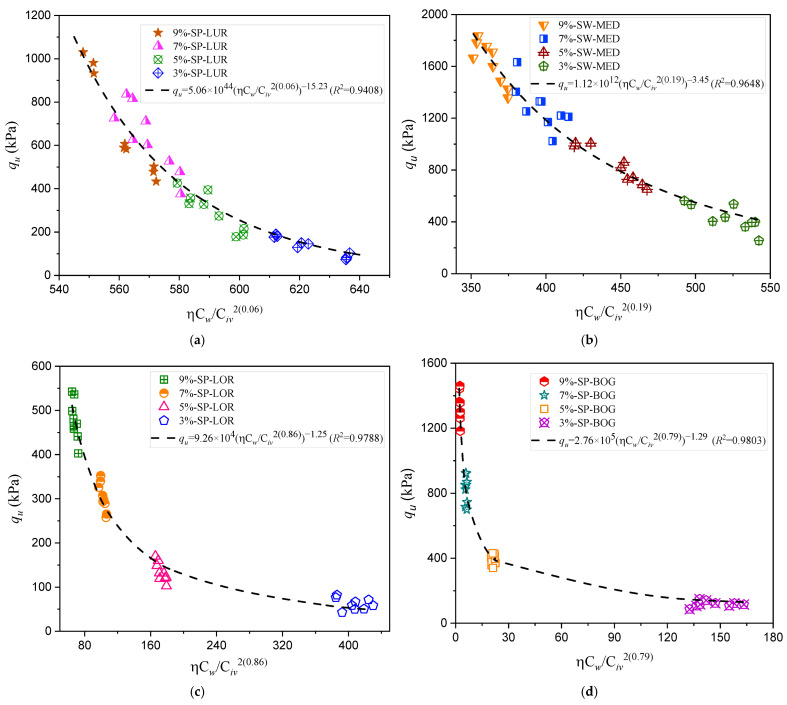
Effects of porosity–water/binder ratio on the unconfined compressive strength of four artificially cemented sands with 9%, 7%, 5%, and 3% dosages of Type III binder cured for seven days: (**a**) Luruaco sand; (**b**) Medellin sand; (**c**) Lorica sand; (**d**) Bogota sand [26].

**Figure 6:**
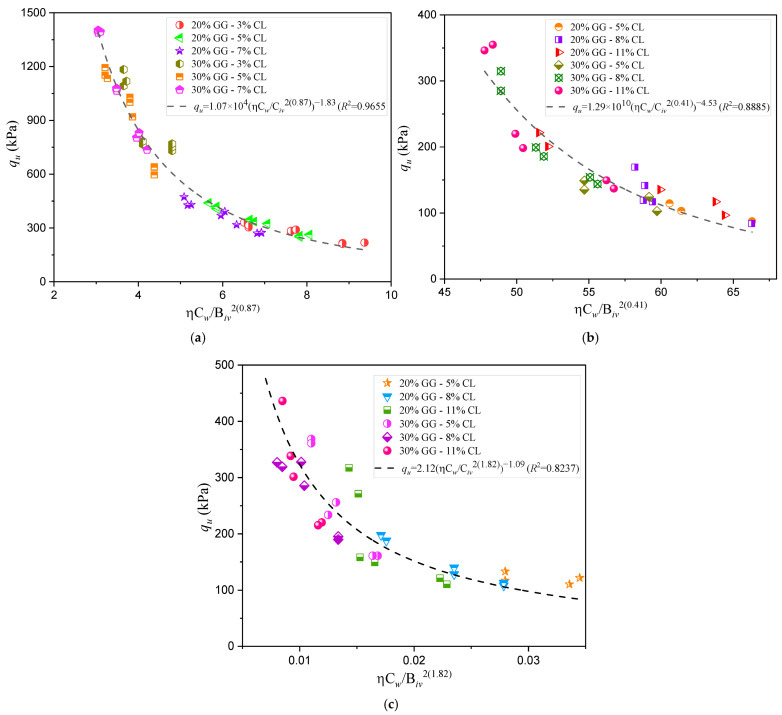
Influence of the porosity–water/binder index on the unconfined compressive strength of compacted blends with carbide lime and glass powder for different sand types: (**a**) Osorio sand; (**b**) Porto Alegre sand; (**c**) Rio Pardo sand [5].

To analyze the efficiency of the new index that incorporates the volumetric water content (Equation (6)) in predicting the compressive strength of sands stabilized with various binders, Figure 5 and Figure 6 present the corresponding adjustments of the potential equation correlating compressive strength with the $\eta C_{w}/B_{iv}$ index. Specifically, Figure 5 illustrates the adjustments obtained for the sand–cement mixtures previously described in Table 2 [26], where the authors applied the index without accounting for the volumetric water content to describe the unconfined compressive strength and stiffness of the studied soil mixtures. Meanwhile, Figure 6 shows the adjustment results for three additional mixtures studied in Brazil, which initially employed the porosity–binder content index to predict unconfined compressive strength. These mixtures include combinations of sand with carbide lime and glass powder. In the present study, the mathematical adjustment of the index was developed by including the water content variable, demonstrating the applicability of the index in stabilization systems with alternative materials [5].

The compressive strength results for the seven stabilized sands using the new porosity–water/binder index $\left(\eta C_{w}/B_{iv}\right)$ are presented in Figure 5a–d and Figure 6a–c, as well as in Table 3. The determination coefficients $\left(R^{2}\right)$ obtained with this index demonstrate high accuracy, ranging from 0.8237 to 0.9803, indicating a strong correlation between the index and the compressive strength of the mixtures. Compared to the original *R*^2^ values, also shown in Table 3, a significant improvement in the model’s predictive capacity is observed, particularly in sands with uniform particle sizes that require higher stabilization levels.

Sands with uniform particle size distribution exhibit reduced particle interaction due to the limited range of sizes, which hinders the natural cohesion of the mixture. This characteristic increases their dependency on binder and water content to achieve adequate strength. The inclusion of water content $\left(C_{w}\right)$ in Equation (5) significantly enhances the model’s accuracy.

The grouping of test points observed in the sands from Lorica (Figure 5c) and Bogotá (Figure 5d) could be influenced by specific conditions during the molding process, such as the dry density used. Regarding the sands from Brazil studied by Consoli et al. [5], it is evident that the traditional index fails to adequately capture the behavior of the new binder, which is a combination of waste glass (GG) and calcium (Ca++, present in carbide lime, CL). This limitation could be related to the fixed values of the scalar parameters a and b. Although the modified index showed significant improvements, some dispersion in the results persists. This suggests that, for this particular combination, both the original and the adjusted index need to incorporate new variables to better represent the mechanical behavior of the cemented mixtures.

For the four Colombian sands, i.e., Luruaco, Medellín, Lorica, and Bogotá, presented in Figure 5 and detailed in Table 3, the results show variations in the fit of the determination coefficients and $C_{iv}$ exponents, reflecting the specific characteristics of each soil. Luruaco sand, with an exponent of 2(0.06), has an R^2^ = 0.9408 with the new index, compared to an original value of 0.9525. This low exponent suggests that the compressive strength of this sand depends less on the binder volume, possibly due to an internal structure that provides resistance without requiring a large amount of binder. This property is characteristic of sands with good particle size distribution, even if they are not well graded, which promotes relative stability without a strong dependence on binder content [7,24].

Similarly, Medellín sand shows a slight decrease in the determination coefficient. The $C_{iv}$ exponent of the binder volume was 2(0.19), indicating that the compressive strength is moderately influenced by the binder volume. This behavior can be explained by the varied grain size and intrinsic properties of Medellín sand, which provide sufficient structural stability, allowing the sand to achieve strength levels without a high dependency on water or binder content [33].

Lorica and Bogotá sands exhibit higher exponents (i.e., 0.86 and 0.79) and are close to unity, reflecting a greater dependency on binder volume to achieve adequate strength. Lorica sand has a $C_{iv}$ exponent of 2(0.86) and an $R^{2}$ = 0.9788 with the new index, slightly improving the original value of 0.9645. Bogotá sand, on the other hand, has an exponent of 2(0.79) and an $R^{2}$ = 0.9803, higher than the original value of 0.9664. This suggests that these sands, with a more uniform gradation that limits particle interaction, benefit from including water content in the index, improving the accuracy of strength prediction [17]. Previous studies, such as those by Horpibulsuk et al. [27], have demonstrated that soils with lower variability in particle size distribution tend to rely more on binder content to achieve strength.

Diambra et al. [17,18] suggest that the exponents x and C in Equation (1) are closely linked to the parameter a, which, in turn, is associated with the soil matrix properties. These exponents exhibit an inverse relationship, where C can reach values up to twice the inverse of a. In certain cases, this relationship may exceed that ratio, indicating that the parametric behavior of sands, such as those from Luruaco, is influenced by the state parameter. Furthermore, in some scenarios, this behavior could be attributed to the presence of fine particles within the material.

For the Brazilian sands shown in Figure 6 and Table 3, the $C_{iv}$ exponents and R^2^ values also exhibit significant variations. Osorio sand, with a $C_{iv}$ exponent of 2(0.87), similar to Lorica sand 2(0.86) (and with both exhibiting an initial adjustment exponent a=1 [5,25]), shows a better fit with the new index ($R^{2}$ = 0.9655) compared to the original value of 0.86. This suggests that, in fine and uniform sandy soils like Osorio and Lorica, the inclusion of water content improves the model’s accuracy. In contrast, Rio Pardo sand has a significantly high $C_{iv}$ exponent 2(1.82) and an $R^{2}$ = 0.8237, compared to an original value of 0.77. This high exponent indicates a strong dependency on binder volume, suggesting a looser internal structure requiring higher binder content to achieve adequate strength [9]. 

Porto Alegre sand, with a $C_{iv}$ exponent of 2(0.41), also shows substantial improvement in the determination coefficient with the new index ($R^{2}$ = 0.8885) compared to the original value of 0.64. This result reflects that water content is a key factor in the stability of this sand, likely due to a particle size distribution that requires additional cohesion provided by the binder in the presence of moisture. Festugato et al. [19] documented that soils with low internal particle interaction or a less dense particle size distribution show significant strength improvements when incorporating water content binders.

According to Baldovino et al. [23], a determination coefficient $\left(R^{2}\right)$ lower than 0.9 could suggest the influence of unknown or unaccounted-for factors in the model that affect the results. These potential factors have not been fully identified or detailed for this study, making further specific analyses necessary for these sands.

Figure 5 and Figure 6 demonstrate that the new porosity–water/binder index is particularly effective in soils with loose structures or dispersed grains, such as Osorio, Porto Alegre, Lorica, and Bogotá sands. In these cases, including water facilitates more significant interactions within the mixture, improving its mechanical behavior. Studies by Baldovino et al. [23,24] have shown that incorporating moisture-related factors into prediction models significantly enhances the accuracy of soil–cement mixtures under various field conditions. This positions the new index as a valuable tool for designing soil stabilizations in diverse soils with different origins and characteristics.

**Figure 7:**
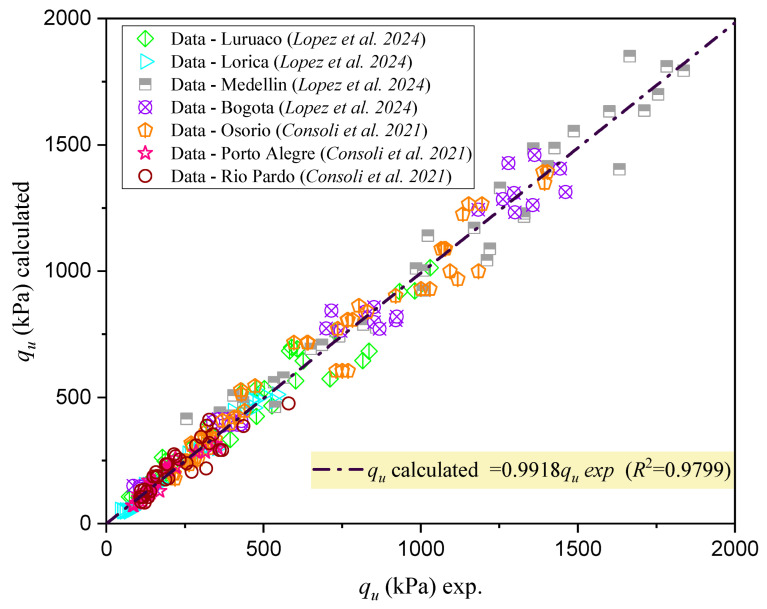
Validation of the estimation of $q_{u}$ for different types of cemented mixtures using the porosity–water/binder index $\left(\eta C_{w}/B_{iv}\right)$ [5,25].

Figure 7 shows that the validation of the unconfined compressive strength $\left(q_{u}\right)$ estimation for different types of cemented mixtures using the porosity–water/binder index $\left(\eta C_{w}/B_{iv}\right)$ demonstrated a strong correlation between calculated and experimental values.

The high $R^{2}$ value (0.9799) indicates excellent predictive accuracy, showing that the model captures the key factors influencing $q_{u}$. The near-unity slope confirms that the calculated $q_{u}$ closely matches experimental results across different mixtures. The constant term suggests a baseline resistance inherent to the materials, likely influenced by factors not directly accounted for in the index. Overall, this validation underscores the index’s reliability and applicability in predicting compressive strength for a wide range of cemented soil mixtures.

### 3.2. Application of the Porosity–Water/Binder Index to Predict the Stiffness of Compacted Blends

**Figure 8:**
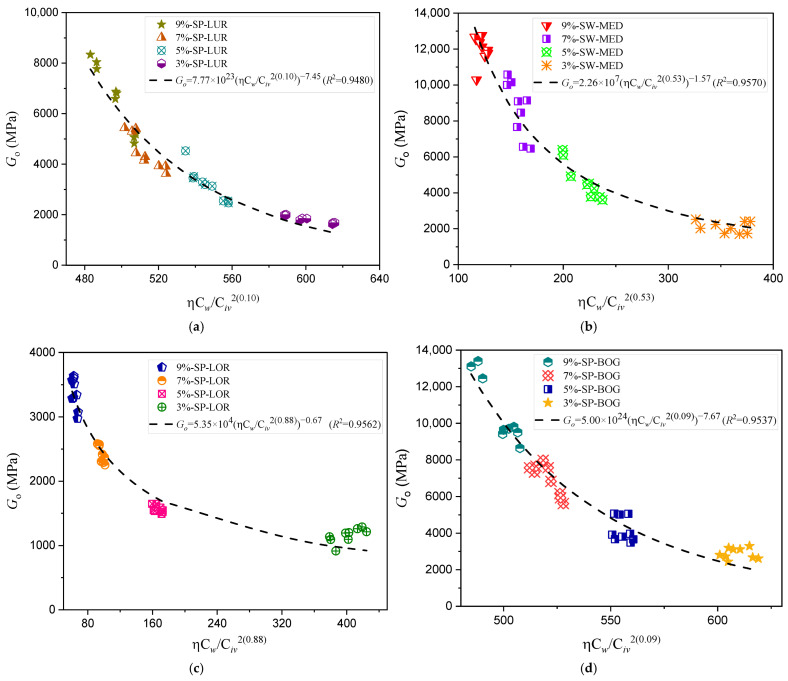
Effects of porosity–water/binder ratio on the stiffness of four artificially cemented sands with 9%, 7%, 5%, and 3% dosages of Type III binder cured for seven days: (**a**) Luruaco sand; (**b**) Medellin sand; (**c**) Lorica sand; (**d**) Bogota sands [25].

**Figure 9:**
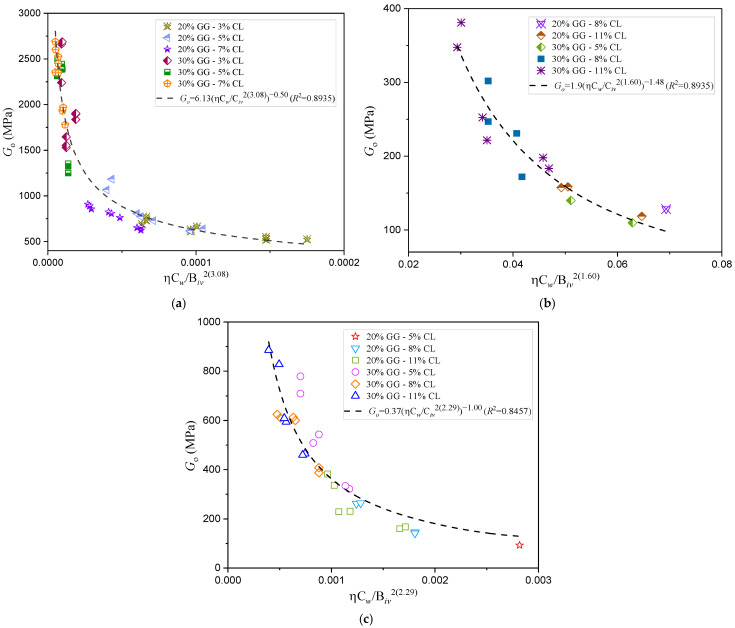
Influence of the porosity–water/binder index on the stiffness of compacted blends with carbide lime and glass powder for different sand types: (**a**) Osorio sand; (**b**) Porto Alegre sand; (**c**) Rio Pardo sand [5].

The results of the ultrasonic pulse velocity tests, detailed in Figure 8 and Figure 9 and Table 4, demonstrate the effectiveness of the porosity–water/binder index $\left(\eta C_{w}/B_{iv}\right)$ in predicting the stiffness of compacted sand–binder mixtures. The index captures the relationship between stiffness $\left(G_{o}\right)$ and the mechanical behavior of these mixtures, where reductions in the porosity-to-binder ratio result in increased stiffness. These findings align with trends observed in unconfined compressive strength analyses, further validating the utility of the index in geotechnical applications involving sands with varying particle size distributions and uniformity [39].

The stiffness of soil mixtures is directly influenced by particle size distribution and uniformity. Sands with a high proportion of fine particles, such as Osorio sand (D50 = 0.16 mm) and Lorica sand (94.1% fine sand), exhibit significantly different behaviors compared to coarser sands, such as Medellín sand (D50 = 1.38 mm) or Porto Alegre sand (32.9% medium sand). Poorly graded sands (SP), like Rio Pardo sand, face challenges due to their low coefficient of uniformity (Cu), which limits particle interlocking, negatively affecting compaction and binder dispersion and resulting in lower stiffness. In contrast, well-graded sands (SW), such as Medellín sand, achieve more efficient particle interlocking thanks to their balanced gradation, enhancing structural stability [33].

The Brazilian sands (i.e., Osorio, Porto Alegre, and Rio Pardo), stabilized with carbide lime and ground glass powder, exhibited a more comprehensive range of stiffness responses, reflecting the challenges of using alternative binders. For instance, Osorio sand, with its high fine sand content (97.6%) and smaller mean particle size (D_50_ = 0.16 mm), presented lower stiffness values compared to Porto Alegre sand, which contains a higher proportion of medium sand (32.9%). Baldovino et al.’s analysis [23,24] supports this trend, indicating that finer particles can hinder binder dispersion, especially in poorly graded sands, unless optimal water content is maintained.

It is important to note that poorly graded sands do not necessarily have less angular particles. Their structural performance is more affected by limited gradation than by particle shape. While angular particles can improve interlocking, the lack of adequate gradation in poorly graded sands hinders the formation of dense and resistant structures. Studies such as those by Chu et al. [40], which analyzed pore pressure generation in poorly graded sands with varying shapes and relative densities, and Ahmed [41], which examined the impact of the coefficient of uniformity on mechanical behavior, emphasize that both gradation and angularity are fundamental for the stiffness and stability of stabilized soils. These findings align with previous research highlighting the influence of granulometric properties on binder dispersion and the formation of stabilized matrices [42,43,44,45].

The stiffness results obtained in the investigation align closely with findings from Baldovino et al. [23], particularly regarding the effectiveness of the porosity–water/binder index $\left(\eta C_{w}/B_{iv}\right)$ as a predictive tool. For the Colombian sands stabilized with cement (Luruaco, Medellín, Lorica, and Bogotá), the index demonstrated strong correlations with stiffness $\left(G_{o}\right)$, with coefficients of determination ($R^{2}$) consistently above 0.94. These results highlight the adaptability of the index in capturing the mechanical behavior of sands with varied granulometric compositions.

For example, Medellín sand, classified as well graded (SW) and exhibiting a higher mean particle size ($D_{50}=1.38$), achieved one of the highest stiffness responses among the Colombian sands. This reflects the influence of its particle arrangement and the uniform distribution of the binder, as supported by Baldovino et al. [24], who emphasized the importance of binder activation in well-graded soils. In contrast, Lorica sand, predominantly composed of fine sand (94.1%), showed a distinct stiffness behavior, requiring precise moisture levels to facilitate uniform binder hydration, also observed in fine-dominated Brazilian sands [23].

The comparison between the coefficients of determination ($R^{2}$) for the new porosity–water/binder index $\left(\eta C_{w}/B_{iv}\right)$ and the original porosity–binder index $\left(\eta /B_{iv}\right)$ reveals essential differences that underscore the improvements introduced by incorporating water content. Across the studied sands, the new $R^{2}$ values demonstrate better alignment with experimental stiffness data, emphasizing the refined predictive capacity of the updated index.

By incorporating the volumetric water content into Equation (5), better curve fitting is achieved for both stiffness and strength, especially in sands with more uniform grain sizes. In addition to this characteristic, another factor contributing to this improvement lies in the treatment of parameter “a”, which in the original index was fixed at 0.28 for Brazilian sands, a value often applied to maintain consistency across different studies [5,8]. However, fixing “a” imposes restrictions on the model’s adaptability, particularly for soils with complex granulometric distributions or those stabilized with alternative binders. This study allowed “a” to vary, resulting in higher coefficients of determination ($R^{2}$) and better alignment with the experimental data.

The fixed value of a = 0.28 used in previous research coincides with the findings of Consoli et al. [7], who demonstrated that a standardized parameter simplifies analysis and ensures replicability in cement-stabilized sands.

**Figure 10:**
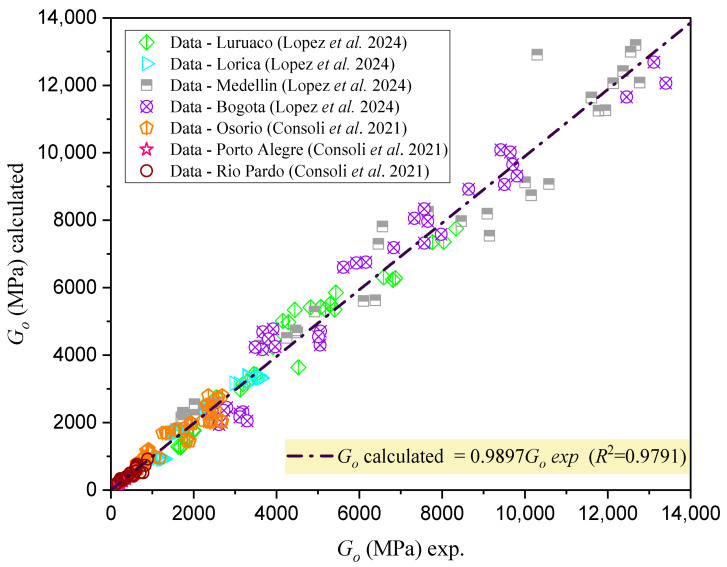
Validation of the estimation of $G_{o}$ for different types of cemented mixtures using the porosity–water/binder index $\left(\eta C_{w}/B_{iv}\right)$ [5,25].

Figure 10 presents a correlation coefficient $R^{2}$ = 0.9791, demonstrating the strong validation of the $G_{o}$ estimation model, highlighting its accuracy and reliability for predicting stiffness in cemented mixtures.

For Colombian sands stabilized with cement, the improvements in $R^{2}$ were less dramatic but still significant. This is mainly because, in the original study [25], the parameter “a” was not fixed since it had not been previously calculated, and the curve fitting was already acceptable, particularly for finer sands like Lorica, where $R^{2}$ increased from 0.9462 to 0.9562. This result underscores the role of water in enhancing binder hydration and distribution [33]. Adjustments made to “a”, such as 0.88 for Lorica sand, reflect the sensitivity of fine granulometry to the interaction between moisture and the binder. Conversely, Medellín sand, classified as well graded (SW), exhibited minimal changes in $R^{2}$, suggesting that its uniform particle size distribution and compaction efficiency allowed the original index to perform adequately.

## 4. Conclusions

This article examines the predictive capacity of the porosity–water/binder content index to estimate the unconfined compressive strength and small-strain stiffness of artificially cemented sands. The analysis encompasses calculating and adjusting the dosage curve for seven previously studied sands: four stabilized with Portland cement and three with a combination of carbide lime and glass powder. Additionally, the parameters and determination coefficients obtained were compared with those adjusted for the porosity/binder content index, highlighting the differences in performance between the two indices. Based on this analysis, the following key conclusions were drawn:

- The porosity–water/binder content index proved to be a highly accurate and reliable tool for predicting the unconfined compressive strength $\left(q_{u}\right)$ and stiffness $\left(G_{o}\right)$ of cemented mixtures with varying characteristics. The $R^{2}$ values being close to unity (0.9799 and 0.9791, respectively) indicates that the model effectively captures the key factors influencing the mechanical behavior of the mixtures.
- This index emerges as a robust alternative for predicting the strength of cemented soils with different binders, achieving determination coefficients near or above 0.9.
- The results suggest that the proposed index may offer superior performance in soils with uniform gradation or loose structures, where water content plays a critical role in the strength and stiffness matrix of the mixtures.
- This index represents a valuable tool for designing cemented mixtures and soil stabilization practices, optimizing binder and water usage. Its application could facilitate more sustainable practices by reducing the dependence on traditional binders, such as Portland cement, and promoting alternative materials.
- For future research, it is recommended to evaluate the feasibility of fixing the value of parameter “a” to simplify the model, considering factors such as soil type, binder used, and specific stabilization conditions.

## Figures and Tables

**Table 1:** Geotechnical properties of the stabilized soils studied.

Sand Detail	Luruaco	Medellín	Lorica	Bogotá	Osorio	Rio Pardo	Porto Alegre
Specific Gravity, Gs	2.73	2.83	2.73	2.69	2.65	2.65	2.68
Gravel (4.75–76.2 mm) (%)	10.8	13.6	-	-	-	-	-
Coarse Sand (2.00–4.75 mm) (%)	20.1	26.3	-	29.5	-	-	-
Medium Sand (0.425–2.00 mm) (%)	42.3	35.5	5.1	69.4	0.3	16.6	32.9
Fine Sand (0.075–0.425 mm (%)	26.6	22.1	94.1	1.0	97.6	81.3	67.1
Silt (0.002–0.0075 mm) (%)	0.2	2.5	0.8	0.1	1.6	2.1	-
Mean Diameter (D_50_, mm)	0.85	1.38	0.24	1.75	0.16	0.25	0.35
USCS Classification	SP	SW	SP	SP	SP	SP	SP
Reference	[25]	[25]	[25]	[25]	[5]	[5]	[5]

**Table 2:** Details of the stabilized soils to apply the efficiency of the proposed porosity–water/binder index.

Type of Mix	Amount of the Additives (%)	% Cement or Lime	Curing Days	Water Content ω (%)	Coefficient of Determination R^2^		Reference
					$\left(q_{u}\right)$	$\left(G_{o}\right)$	
Luruaco: sand	-	3, 5, 7 and 9 *	7	10	0.95	0.91	[25]
Medellín: sand	-	3, 5, 7 and 9 *	7	10	0.96	0.95	[25]
Lorica: sand	-	3, 5, 7 and 9 *	7	10	0.98	0.96	[25]
Bogotá: sand	-	3, 5, 7 and 9 *	7	10	0.97	0.94	[25]
Osorio: sand–carbide lime–glass powder	20 and 30	3, 5 and 7 **	7	11	0.86	0.73	[5]
Porto Alegre: sand-carbide lime–glass powder	20 and 30	5, 8 and 11 **	7	11	0.64	0.41	[5]
Rio Pardo: sand–carbide lime–glass powder	20 and 30	5, 8 and 11 **	7	11	0.77	0.31	[5]

* Stabilized with Portland cement type III, ** carbide lime.

**Table 3:** Compressive strength equation of compacted blends.

Type of Mix	Compressive Strength Equation	New Coefficient of Determination	Original Coefficient of Determination
Luruaco sand–cement	$q_{u}=5.06\times 10^{44}{\left(\frac{\eta C_{w}}{C_{iv}^{2\left(0.06\right)}}\right)}^{-15.23}$	$R^{2}=0.9408$	$R^{2}=0.9525$
Medellín sand–cement	$q_{u}=1.12\times 10^{12}{\left(\frac{\eta C_{w}}{C_{iv}^{2\left(0.19\right)}}\right)}^{-3.45}$	$R^{2}=0.9648$	$R^{2}=0.9792$
Lorica sand–cement	$q_{u}=9.26\times 10^{4}{\left(\frac{\eta C_{w}}{C_{iv}^{2\left(0.86\right)}}\right)}^{-1.35}$	$R^{2}=0.9788$	$R^{2}=0.9645$
Bogotá sand–cement	$q_{u}=2.76\times 10^{5}{\left(\frac{\eta C_{w}}{C_{iv}^{2\left(0.79\right)}}\right)}^{-1.29}$	$R^{2}=0.9803$	$R^{2}=0.9664$
Osorio sand–carbide lime–glass powder	$q_{u}=1.07\times 10^{4}{\left(\frac{\eta C_{w}}{B_{iv}^{2\left(0.87\right)}}\right)}^{-1.83}$	$R^{2}=0.9655$	$R^{2}=0.86\;*$
Porto Alegre sand–carbide lime–glass powder	$q_{u}=1.29\times 10^{10}{\left(\frac{\eta C_{w}}{B_{iv}^{2\left(0.41\right)}}\right)}^{-4.53}$	$R^{2}=0.8885$	$R^{2}=0.64\;*$
Rio Pardo sand–carbide lime–glass powder	$q_{u}=2.12{\left(\frac{\eta C_{w}}{B_{iv}^{2\left(1.82\right)}}\right)}^{-1.09}$	$R^{2}=0.8237$	$R^{2}=0.77\;*$

* Coefficient of Determination (R^2^) calculated with two decimals.

**Table 4:** Stiffness equation of compacted blends.

Type of Mix	Stiffness Equation	New Coefficient of Determination	Original Coefficient of Determination
Luruaco sand–cement	$G_{0}=7.77\times 10^{23}{\left(\frac{\eta C_{w}}{C_{iv}^{2\left(0.10\right)}}\right)}^{-7.45}$	$R^{2}=0.9480$	$R^{2}=0.9138$
Medellín sand–cement	$G_{0}=2.26\times 10^{7}{\left(\frac{\eta C_{w}}{C_{iv}^{2\left(0.53\right)}}\right)}^{-1.57}$	$R^{2}=0.9570$	$R^{2}=0.9557$
Lorica sand–cement	$G_{0}=5.53\times 10^{4}{\left(\frac{\eta C_{w}}{C_{iv}^{2\left(0.88\right)}}\right)}^{-0.67}$	$R^{2}=0.9562$	$R^{2}=0.942$
Bogotá sand–cement	$G_{0}=5.00\times 10^{24}{\left(\frac{\eta C_{w}}{C_{iv}^{2\left(0.09\right)}}\right)}^{-7.67}$	$R^{2}=0.9537$	$R^{2}=0.9363$
Osorio sand–carbide lime–glass powder	$G_{0}=6.13{\left(\frac{\eta C_{w}}{B_{iv}^{2\left(3.08\right)}}\right)}^{-0.5}$	$R^{2}=0.8935$	$R^{2}=0.73\;*$
Porto Alegre sand–carbide lime–glass powder	$G_{0}=1.9{\left(\frac{\eta C_{w}}{B_{iv}^{2\left(1.60\right)}}\right)}^{-1.48}$	$R^{2}=0.8935$	$R^{2}=0.41\;*$
Rio Pardo sand–carbide lime–glass powder	$G_{0}=0.37{\left(\frac{\eta C_{w}}{B_{iv}^{2\left(2.29\right)}}\right)}^{-1.00}$	$R^{2}=0.8457$	$R^{2}=0.31\;*$

* Coefficient of Determination (R^2^) calculated with two decimals.

## Data Availability

The original contributions presented in this study are included in the article. Further inquiries can be directed to the corresponding authors.
